# Systemic Administration of Acazicolcept, a Dual CD28 and Inducible T cell Costimulator Inhibitor, Ameliorates Experimental Autoimmune Uveitis

**DOI:** 10.1167/tvst.12.3.27

**Published:** 2023-03-28

**Authors:** Leslie Wilson, Katherine E. Lewis, Lawrence S. Evans, Stacey R. Dillon, Kathryn L. Pepple

**Affiliations:** 1 Department of Ophthalmology, University of Washington, Seattle, WA, USA; 2 Roger and Angie Karalis Johnson Retina Center, University of Washington School of Medicine, Seattle, WA, USA; 3 Discovery Research, Alpine Immune Sciences, Seattle, WA, USA; 4 Translational Medicine, Alpine Immune Sciences, Seattle, WA, USA

**Keywords:** uveitis, ocular inflammation, costimulation inhibition, T cell, autoimmunity, inducible T cell costimulator (ICOS), CD28

## Abstract

**Purpose:**

Combined inhibition of CD28 and inducible T cell costimulator (ICOS) pathways with acazicolcept (ALPN-101) represents a potential new treatment for uveitis. Here, we evaluate preclinical efficacy using experimental autoimmune uveitis (EAU) in Lewis rats.

**Methods:**

Efficacy was tested in 57 Lewis rats treated with either systemic (subcutaneous) or local (intravitreal) administration of acazicolcept and compared to treatment with a matched Fc-only control or corticosteroid. Impact of treatment on uveitis was assessed using clinical scoring, optical coherence tomography (OCT), and histology. Ocular effector T cell populations were determined using flow cytometry, and multiplex ELISA used to measure aqueous cytokine concentrations.

**Results:**

When compared to Fc control treatment, systemic acazicolcept led to statistically significant decreases in clinical score (*P* < 0.01), histologic score (*P* < 0.05), and number of ocular CD45+ cells (*P* < 0.01). Number of ocular CD4+ and CD8+ T cells expressing IL-17A+ and IFNγ+ were also decreased with statistical significance (*P* < 0.01). Similar results were achieved with corticosteroids. Intravitreal acazicolcept decreased inflammation scores when compared to untreated fellow eyes and to Fc control treated eyes, although not statistically significant. Systemic toxicity, measured by weight loss, occurred in the corticosteroid-treated, but not in the acazicolcept-treated animals.

**Conclusions:**

Systemic treatment with acazicolcept statistically significantly suppressed EAU. Acazicolcept was well-tolerated without the weight loss associated with corticosteroids. Acazicolcept may be an effective alternative to corticosteroids for use in treating autoimmune uveitis. Additional studies are needed to clarify the optimal dose and route for use in humans.

**Translational Relevance:**

We show that T cell costimulatory blockade could be an effective mechanism for treating uveitis.

## Introduction

Uveitis is an inflammatory disorder of the eye that ranks among the top 10 causes of visual disability and blindness in the developed world.^1^^–^^4^ Vision loss results from direct inflammatory damage of ocular structures, or from complications, such as macular edema, cataract, and glaucoma.^5^^,^^6^ However, when noninfectious uveitis is treated appropriately, vision loss is largely preventable.^7^^,^^8^ Corticosteroids have historically been the mainstay of uveitis treatment, but their long-term use is limited by ocular and systemic adverse effects.^9^^–^^11^ Alternative options for corticosteroid sparing therapy include using nonspecific immune suppressants^12^ or targeted therapy with anti-tumor necrosis factor alpha (TNFα) biologics^13^^–^^15^ but the efficacy of these medications is suboptimal, and rescue therapy with corticosteroids can be required in up to 25% to 40% of patients.^16^^–^^21^ Development of a new therapy with a novel mechanism of action would be an important advance for patients with refractory disease. Additionally, a locally administered treatment could provide patients with a better tolerated option for disease control.^22^

Experimental autoimmune uveitis (EAU) is a well-established model of uveitis and has been used extensively in preclinical testing of novel therapies.^23^ EAU is induced by immunization with specific retinal proteins or peptides, and disease is mediated by T helper type 1 (Th1) and T helper type 17 (Th17) mechanisms.^24^ Activation, proliferation, and differentiation of naïve T cells into these pathogenic T helper subsets require antigen-specific recognition of the autoantigen as well as the interaction of T cell costimulatory receptors with their ligands on antigen presenting cells (APCs).^25^^,^^26^ CD28 and inducible T cell costimulator (ICOS) are 2 T cell surface receptors in the immunoglobulin superfamily that play partially overlapping roles in T cell costimulation; CD28 binds its counter receptors CD80 and CD86 on APCs, whereas ICOS binds to ICOS ligand (ICOSL).^27^^,^^28^ The costimulatory receptor CD28 is constitutively expressed on naïve and memory T cells, and is required for T cell activation, proliferation, and survival.^29^^–^^31^ In the absence of CD28, CD4+ T cell responses to infection and immunization are diminished, with a failure of Th1 and T follicular helper (Tfh) development.^32^ ICOS, on the other hand, is not expressed on naïve T cells but is rapidly upregulated upon their activation.^33^^,^^34^ Signaling through ICOS augments T cell proliferation and plays a non-redundant role in the development of Th17 and Tfh cells.^35^ Previous work rodent models of EAU has shown that ICOS is expressed on ocular infiltrating CD4+ T cells and that blockade of either CD28 or ICOS effectively decreased disease severity if administered during disease induction, but not during active disease.^25^^,^^36^^–^^38^ Together, these data indicate that T costimulatory pathways are mechanistically important in EAU, but that combined costimulatory blockade may be needed to achieve clinically relevant disease control.

Acazicolcept (ALPN-101) is a novel Fc fusion protein engineered to simultaneously inhibit both the CD28 and ICOS costimulatory pathways. Dual targeting was achieved through directed evolution of the ICOSL extracellular domain, and creation of an ICOSL variant Ig domain (vIgD) that binds to both ICOS and CD28 with high affinity.^39^ The therapeutic candidate acazicolcept is a dimer of the ICOSL vIgD fused to a modified IgG1 Fc that does not bind FcγR (i.e. CD16a, CD32, or CD64) or complement component 1q (C1q) but does bind FcRn. Preclinical studies in mice have shown that acazicolcept effectively suppresses disease activity in models of autoimmune arthritis, systemic sclerosis, Sjögren's syndrome, and graft versus host disease.^40^^–^^43^ We therefore sought to demonstrate the efficacy of dual T cell costimulatory blockade on controlling uveitis following either systemic or local administration of acazicolcept in the Lewis rat model of EAU.

## Methods

### Animals, Experimental Autoimmune Uveitis Induction, and Study Treatments

This animal study protocol was approved by the Animal Care and Use Committee of the University of Washington (animal study protocol # 4481-01) and was compliant with the ARVO Statement for the Use of Animals in Ophthalmic and Vision Research. Fifty-seven female Lewis rats 6 to 8 weeks of age (body weights approximately 125–149 g) were purchased (Envigo, Somerset, NJ, USA) and maintained with standard chow and water ad libitum under specific pathogen-free conditions. EAU was generated with subcutaneous injection of 60 µg interphotoreceptor retinoid binding protein peptide R16 (ADGSSWEGVGVVPDV; Peptide 2.0, Chantilly, VA, USA) in complete Freund's adjuvant (Difco # 263910) in 2 divided doses to each hip.^44^

Systemic treatment was administered by subcutaneous (s.c.) injection between the shoulder blades on day 7 after induction of EAU in 10 rats per treatment group (total *n* = 30). The doses administered were 100 mg/kg acazicolcept (ALPN-101), 50 mg/kg Fc control, and 0.8 mg/kg triamcinolone acetate (steroid). Acazicolcept (ALPN-101; ICOSL vIgD-Fc; 80.8 kDa), was provided by Alpine Immune Sciences (AIS) (Seattle, WA, USA)^41^ and produced at KBI Biopharma (Durham, NC, USA). The matched Fc control protein (51.8 kDa) was produced at AIS. For the local treatment study, intravitreal injections were performed on the right eye on days 8 and 11 after induction of EAU. The interval between intravitreal doses was determined by pharmacokinetic data obtained in a pilot study demonstrating acazicolcept drug levels were maintained above target range in the eye for at least 48 hours after administration (data not shown). Nine animals per treatment group (total *n* = 27) were injected twice with 3 µl of either 80 µg acazicolcept (ALPN-101), 40 µg Fc control, or 100 µg triamcinolone acetonide (steroid). Injections were performed as previously reported on anesthetized animals using sterile practices under a dissection microscope by a single surgeon (author K.P.).^44^

### Clinical Scoring, Optical Coherence Tomography System, Image Acquisition, and Analysis

Clinical scores were performed by a masked grader (author L.W.) using an established scale.^23^ OCT images were acquired using the Bioptigen Envisu R2300, as previously described.^36^ Briefly, anterior segment volume scans centered on the corneal apex covering an area of 5 mm × 5 mm (1000 Ascan/Bscan × 200 B-scans) were captured using a Bioptigen 18 mm telecentric lens (product # 90-BORE-G3-18; Bioptigen, Inc., Morrisville, NC, USA). A single grader (author K.P.), masked to eye, treatment, and experimental day, scored the degree of inflammation using an established and validated OCT imaging score system.^45^

### Postmortem Aqueous Humor Analysis, Flow Cytometry, and Histology

On day 14, animals were euthanized and their eyes collected for either histology or combined aqueous humor cytokine analysis and flow cytometry. For histology, whole eyes were fixed in 10% neutral buffered formalin (Sigma-Aldrich Corp., St. Louis, MO, USA) for at least 24 hours. Paraffin block sections (4 µm) were stained with hematoxylin and eosin (H&E) and scored by a single grader (author K.P.), masked to treatment using an established grading system.^46^ Three sections per eye were scored and averaged. For animals receiving systemic treatment, final score was assigned per animal and calculated as the average score of both eyes.

Aqueous humor was collected in an ethylenediaminetetraacetic acid (EDTA)-containing capillary tube (Sarstedt, Nümbrecht, Germany) after corneal paracentesis with a 30-gauge needle (Becton, Dickinson and Company, Franklin Lakes, NJ, USA). Ten to 15 µL of aqueous was collected from each eye and stored at −80°C, in combination with 1X protease inhibitor (Sigma-Aldrich Corp.), until assayed. Aqueous protein was quantified using Pierce 660 nm Protein Assay Reagent (Thermo Fisher Scientific, Madison, WI, USA) for colorimetric detection on the Nanodrop ND-1000 spectrophotometer (Thermo Fisher Scientific); 1 µL aqueous was used for total protein concentration determination. The remaining aqueous (9–14 µL) was diluted in an equal volume of radioimmunoprecipitation assay (RIPA) buffer containing phenylmethylsulfonyl fluoride (PMSF) and protease inhibitor cocktail, according to the manufacturer's protocol, and divided equally for testing in duplicate. For cytokine analysis in the systemic treatment study, aqueous from the right and left eye of the same animal were pooled into a single sample (16–36 µL per sample). Aqueous from the local treatment study was not pooled. Aqueous cytokine concentrations were determined using the Milliplex MAP rat cytokine/chemokine premixed 27-plex immunology multiplex assay (EMD Millipore Corp., Billerica, MA, USA). The cytokines measured were granulocyte-colony stimulating factor (G-CSF), eotaxin (CCL11), granulocyte monocyte-colony stimulating factor (GM-CSF), IL-1α, macrophage inflammatory protein-1α (MIP-1α/CCL3), IL-2, epidermal growth factor (EGF), IL-13, IL-12p70, IL-5, monocyte chemoattractant protein-1 (MCP-1/CCL2), IFNγ–induced protein 10 (IP-10/CXCL10), fractalkine (CX3CL1), lipopolysaccharide-induced CXC chemokine (LIX/CXCL5), MIP-2 (CXCL2), leptin, IL-4, IL-6, IL-10, IFN-γ, IL-17A, IL-18, growth-related oncogene/keratinocyte chemokine (GRO/KC/CXCL1), VEGF, TNFα, and regulated on activation, normal T cell expressed and secreted (RANTES/CCL5). Due to failure of the GRO/KC controls, results for this cytokine are not reported. Samples were analyzed using the MAGPIX system (Luminex, Austin, TX, USA) with xPonent software version 4.2 (EMD Millipore). Data analysis was performed using Milliplex Analyst Standard version 5.2 software (Vigenetech). Statistics analysis and graphing was performed using GraphPad Prism 9.0 software (GraphPad Software, La Jolla, CA, USA).

For flow cytometry analysis, the retina, retinal pigmented epithelium (RPE), choroid, and vitreous were removed from the eyes following aspiration of the aqueous (for cytokine analysis) and collected in 1X phosphate-buffered saline (PBS) with 2% fetal bovine serum (FBS; Cytiva, Marlborough, MA, USA). A single cell suspension was generated, as described previously,^44^ with mechanical disruption using microsurgical scissors, followed by digestion with Collagenase D (Roche, Switzerland) at 37 degrees Celsius (°C), and filtration through 70 mm mesh (Elko Filtering Co., Switzerland). Live cell counting was performed using acridine orange (AO)/propidium iodide (PI) and a Nexcelom cell counter (Auto2000). For cell surface marker identification, cells were stained with live-dead blue viability dye (Invitrogen; 1:1000), following by anti-human IgG (BioLegend [BL] 409318; 1:100), and antibodies to the following rat markers: CD28 (Becton Dickinson [BD] 742583; 1:100), CD3 (BD 563949; 1:100), ICOS (BL 313534; 1:100), CD25 (BL 202103; 1:100), CD11b/c (BL 201820; 1:100), RT1B (BD 205308; 1:200), CD4 (BL 201516; 1:100), FoxP3 (BL 320014; 1:40), CD8 (BD 741771; 1:100), and CD45 (BL 202216; 1:100). For intracellular cytokine staining, cells were incubated with phorbol 12-myristate 13-acetate (PMA) and ionomycin with Golgi block for 4 hours at 37°C prior to staining with live-dead blue viability dye (Invitrogen; 1:1000), and antibodies against rat markers CD8 (BD 741771; 1:100), CD3 (BD 563949; 1:100), CD4 (BL 201516; 1:100), CD45 (BL 202216; 1:100), IL-17A (eBiosciences 11-7177-81; 1:40), and IFNγ (BL 507806; 1:20). For samples in the systemic treatment study, right and left eye samples from each animal were pooled prior to staining and divided into two samples for surface and intracellular staining. For the local treatment study, samples were not pooled. Stained samples were collected on a Beckman Coulter Cytoflex LX or a BD LSRII flow cytometer and data analyzed using FlowJo software (version 10.7.1; Ashland, OR, USA).

### Statistical Analysis

Statistical analysis and graphing were performed using GraphPad Prism 9.3 software (GraphPad Software, La Jolla, CA, USA). For normally distributed data, analysis was performed using 1-way ANOVA with Tukey's multiple comparisons test or Brown-Forsythe and Welch's ANOVA with Dunnett's T3 multiple comparison. For nonparametric data, Kruskal-Wallis with Dunn's multiple comparisons test was used. Corrected *P* values < 0.05 were considered statistically significant.

## Results

### Systemic Treatment With Acazicolcept Decreases Clinical and Histologic Scores in EAU Similar to Treatment With Corticosteroids

**Figure 1.**
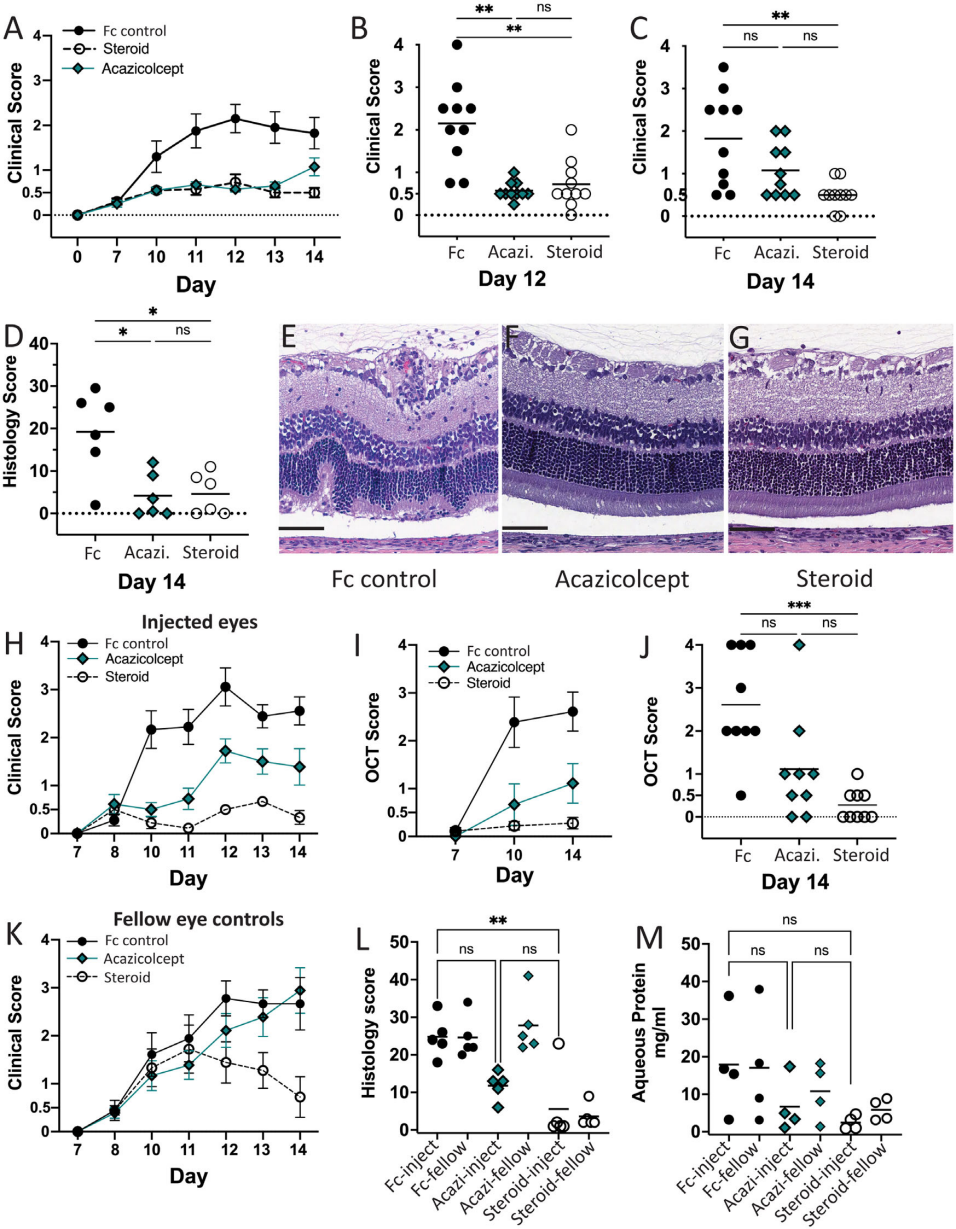
**Systemic and local administration of acazicolcept decreases ocular inflammation in EAU.** (**A**) Systemic treatment study average clinical score by day. Error bars = SEM. (**B**) Systemic treatment day 12 and (**C**) day 14 clinical scores by animal. Mean score per treatment group indicated by the bar. (**D**) Systemic treatment day 14 histology score. (**E**) H&E stain of retina from systemic Fc control-treated animals with vitritis, retinal folds, outer segment loss, and retinal, subretinal, and perivascular infiltration. (**F**) Systemic acazicolcept- and (**G**) corticosteroid-treated retina with preserved architecture. Intravitreal treatment study (**H**) clinical and (**I**) OCT scores of treated eyes. (**J**) Intravitreal study day 14 OCT scores by treated eye. (**K**) Average clinical score of uninjected (control) eyes from intravitreal study. Scores increased in Fc control and acazicolcept animals consistent with local function. In contrast, scores decreased in fellow eyes of animals treated with intravitreal corticosteroid injections indicating systemic impacts following local injection. (**L**) Histology scores and (**M**) aqueous protein concentration from treated and uninjected (control) eyes from intravitreal study animals. Comparisons by Kruskal-Wallis test with Dunn's multiple comparisons. *P < 0.05, **P ≤ 0.01, ns = *P* ≥ 0.05.

To test the efficacy of systemic administration of acazicolcept in the control of ocular inflammation, EAU was induced in 30 Lewis rats, and treatment was administered 7 days later by subcutaneous injection of triamcinolone (steroid control), acazicolcept, or a matched inert Fc control. In the Lewis rat model of EAU, inflammation scores reached peak severity (≥2) starting on day 12 and then plateaued or decreased slightly by the study end point at day 14.^23^^,^^47^ This pattern was observed in the Fc control-treated animals, whereas acazicolcept- and corticosteroid-treated animals demonstrated low scores throughout the study (Fig. 1A). A comparison of clinical scores on day 12 showed average clinical scores of both acazicolcept- (0.58 ± 0.21, *P* < 0.01) and corticosteroid (0.73 ± 0.57, *P* < 0.01) -treated animals were statistically significantly lower than Fc control (2.15 ± 0.99) -treated animals (Fig. 1B). Subsequent comparison on day 14 (Fig. 1C) demonstrated that although acazicolcept-treated animals continued to show lower average clinical inflammation scores when compared to Fc control-treated animals, the difference was no longer statistically significant (1.07 ± 0.64 vs. 1.83 ± 1.11, *P* = 0.17). Corticosteroid treated animals on day 14 continued to demonstrate statistically significantly lower clinical scores than Fc control treated animals (0.5 ± 0.33, *P* < 0.01). On the other hand, when the disease burden over the course of the study period for each animal was analyzed by the area under the curve (AUC), the median of the acazicolcept- (4.625) and corticosteroid-treated (4.500) groups each had a statistically significantly lower median AUC as compared to the Fc control group (10.13; *P* = 0.0100 and *P* = 0.0047, respectively). Consistent with clinical findings that systemic treatment decreased inflammation, mean histology scores following acazicolcept (4.2 ± 5.2, *P* < 0.05) and corticosteroid (4.6 ± 4.8, *P* < 0.05) treatment were also statistically significantly lower than with Fc control (19.3 ± 10.0; Fig. 1D). Signs of retinal damage typical to EAU, including retinal folds, retinal vasculitis, and photoreceptor outer segment loss and inflammatory cell infiltration, (Fig. 1E) were not seen with systemic acazicolcept (Fig. 1F) or corticosteroid (Fig. 1G) treatment. However, signs of mild inflammation, including few inflammatory cells in the aqueous and vitreous, were present in the eyes of animals treated with either systemic acazicolcept or corticosteroids (data not shown).

Local administration is a desired alternative for patients with uveitis due to the potential risk of side effects from systemic treatment with corticosteroids or other immunosuppressants. Thus, efficacy of administration of acazicolcept by intravitreal injection was tested. On days 8 and 11 after EAU induction, the right eye was injected with acazicolcept, triamcinolone (steroid control), or Fc control. Prior to injection on day 8, there was no difference in average clinical inflammation score between treatment groups (Fig. 1H). However, on day 10 when inflammation was measured using both clinical score and OCT score (Fig. 1I), acazicolcept-treated eyes had statistically significantly lower average clinical (0.5 ± 0.43, *P* < 0.05) and OCT (0.67 ± 1.3, *P* < 0.05) scores than Fc control-treated eyes (clinical = 2.17 ± 1.73 and OCT = 2.39 ± 1.56). In corticosteroid-treated eyes, clinical scores (0.22 ± 0.36, *P* < 0.05) and OCT scores (0.22 ± 0.26, *P* < 0.001) were also statistically significantly lower than in Fc control eyes, but the differences between the acazicolcept- and corticosteroid-treated eyes were not.

Following the second intravitreal injection performed on day 11, clinical scores increased on day 12 across all treatment groups, suggesting a possible impact of repeat trauma on inflammation. By study day 14, acazicolcept-treated eyes continued to show lower average clinical (1.39 ± 1.14, *P* = 0.18) and OCT scores (1.11 ± 1.24, *P* = 0.11) than Fc control-treated eyes (clinical = 2.56 ± 0.88 and OCT = 2.61 ± 1.22), but the differences were no longer statistically significant. In contrast, scores in corticosteroid-treated eyes were statistically significantly decreased when compared to Fc control-treated eyes (clinical = 0.33 ± 0.43 and OCT = 0.27 ± 0.36, both *P* < 0.001; Fig. 1J). Interestingly, the clinical scores of the uninjected fellow eyes (Fig. 1K) of corticosteroid-treated animals trended down sharply after the second injection on day 10, and by day 14 were statistically significantly lower than the contralateral eyes of both acazicolcept- and Fc control-treated eyes (*P* < 0.05). This impact of intravitreal corticosteroids on the contralateral eye suggests that the cumulative dose of triamcinolone delivered locally was sufficient to suppress inflammation systemically. In contrast, the effect of acazicolcept was more likely mediated locally as the contralateral (control) eyes did not show decreased clinical (see Fig. 1K) or histology (Fig. 1L) scores. Histology scores of acazicolcept-treated eyes (11.8 ± 3.7, *P* = 0.13) were lower than those for Fc control-treated eyes (24.8 ± 5.5), although not statistically significant, whereas corticosteroid treatment decreased inflammation in both treated (5.6 ± 9.7, *P* < 0.01) and uninjected control eyes (3.6 ± 3.0; see Fig. 1L). As a final measure of inflammation severity, aqueous protein concentration was compared among the injected eyes of four animals per treatment group. Consistent with prior studies showing aqueous protein concentration increases with inflammation severity,^48^^,^^49^ average protein concentrations were highest in Fc control-treated eyes (17.9 mg/mL), with little difference between injected and fellow (control) eyes of the same animal (Fig. 1M). Although average aqueous protein concentrations were lower in both acazicolcept- (6.7 mg/mL, *P* > 0.9) and corticosteroid-treated eyes (2.4 mg/mL, *P* = 0.15), the difference when compared to Fc control eyes was not statistically significant. Overall, the anti-inflammatory benefit of local administration of acazicolcept was not equivalent to the benefit from systemic administration. However, injected eyes did show a trend of decreased inflammation when compared to untreated fellow eyes of the same animal for each of the day 14 outcome measures (Supplementary Fig. S1).

### Systemic Treatment With Acazicolcept Statistically Significantly Decreases Ocular Infiltration of Pathogenic T cells

**Figure 2.**
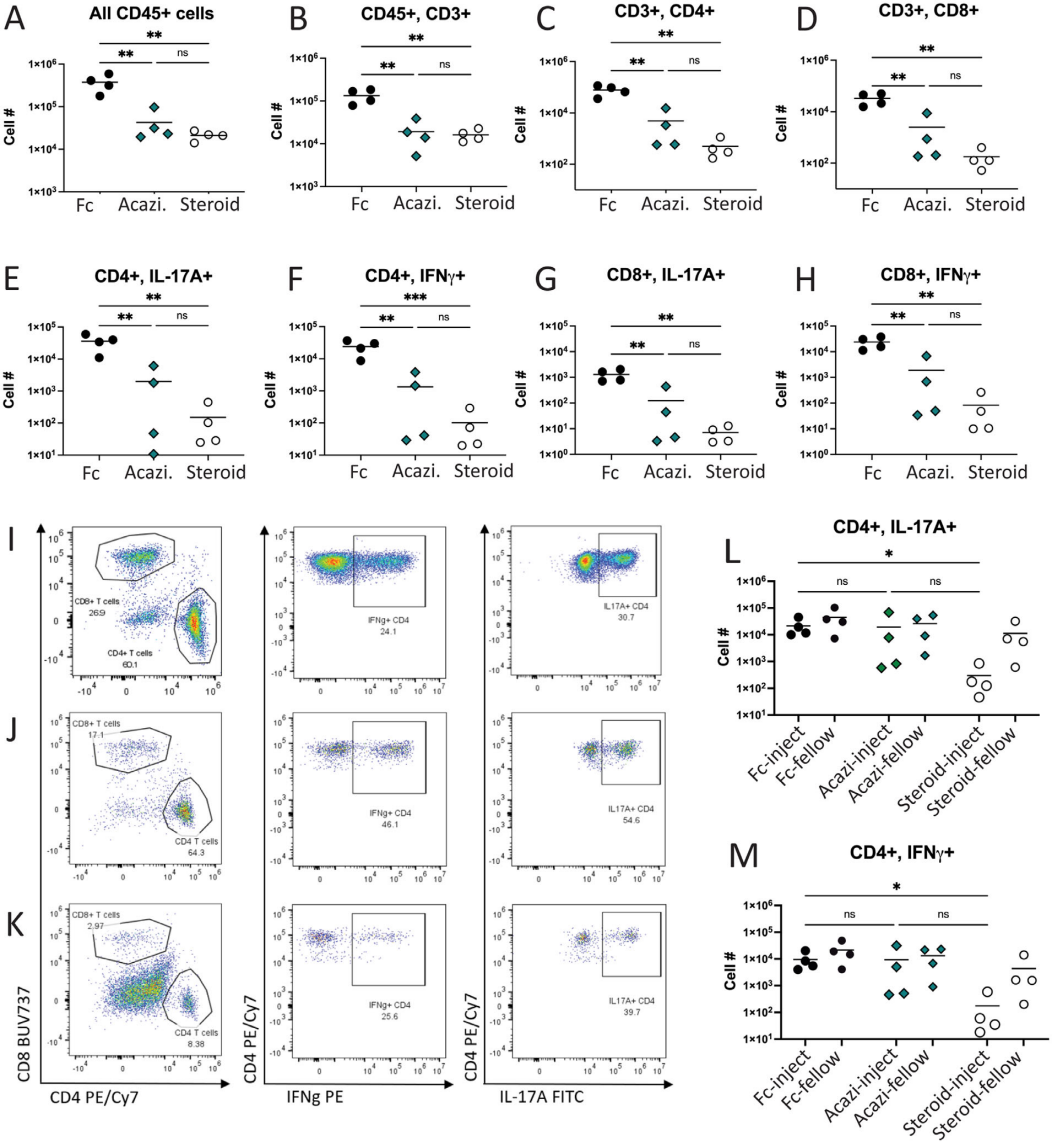
**Systemic administration of acazicolcept and triamcinolone statistically significantly decreases the number of ocular infiltrating IL-17A and IFNγ expressing CD4+ T cells.** Flow cytometry was performed on intraocular contents of both eyes (pooled) from four animals in the systemic treatment study (**A-K**) or from individual injected and fellow control eyes from four animals in the local injection study (**L,** **M**). (**A-H**) Quantitative results from eyes in the systemic treatment study arm. All samples were first gated for single, live CD45+ events. Results are shown as total cell number per sample, mean of all samples indicated by the bar. (**I-K**) Sample gates from one animal per treatment group in the systemic administration arm demonstrating the ocular CD8+ and CD4+ populations as well as expression of IFNγ or IL-17 in the CD4+ population. Population percentages indicated on the graph. (**I**) Fc control treatment; (**J**) acazicolcept treatment; (**K**) corticosteroid treatment. Comparisons performed by one-way ANOVA with Tukey's multiple comparisons test. *P < 0.05, **P < 0.01, ***P < 0.001, ns = *P* ≥ 0.05.

To evaluate the impact of treatment on the local inflammatory cell infiltrate**,** ocular contents were analyzed by flow cytometry on day 14 (Fig. 2, Supplementary Fig. S2). In the systemic treatment study, average ocular CD45+ cell number was statistically significantly lower following subcutaneous administration of acazicolcept (5.46 × 10^4^ cells/eye, *P* < 0.05) or corticosteroid (1.53 × 10^4^ cells/eye, *P* < 0.01) when compared to Fc control (28.3 × 10^4^ cells/eye; see Fig. 2A). The number of ocular CD3+ T cells was also statistically significantly decreased by systemic acazicolcept (2.03 × 10^4^ cells/eye, *P* < 0.01) or corticosteroid (1.03 × 10^4^ cells/eye, *P* < 0.01) treatment when compared to Fc control (10.7 × 10^4^ cells/eye; see Figs. 2B, 2C, 2D). Consistent with prior studies in EAU, a large proportion of CD4+ T cells in Fc control-treated eyes expressed either intracellular IL-17A (44%) or IFNγ (26%). The number of intraocular CD4+ T cells expressing these cytokines was statistically significantly lower following systemic treatment with either acazicolcept or corticosteroid (Figs. 2E, 2F). A similar, significant decrease in the number of ocular CD8+ T cells expressing IL-17A and IFNγ occurred following systemic treatment with acazicolcept or corticosteroid (Figs. 2G, 2H). In contrast, intravitreal injection of acazicolcept did not statistically significantly decrease ocular T cell numbers or cytokine expression compared to Fc control treatment (Figs. 2I, 2J) or when compared to uninjected fellow eyes. Only local treatment with corticosteroids provided a statistically significant decrease (*P* < 0.05) in the number of pathogenic T cells infiltrating the eye in EAU. Together, these data demonstrate a statistically significant treatment effect at the level of the pathogenic ocular T cell populations in EAU following systemic, but not local, treatment with acazicolcept.

**Figure 3.**
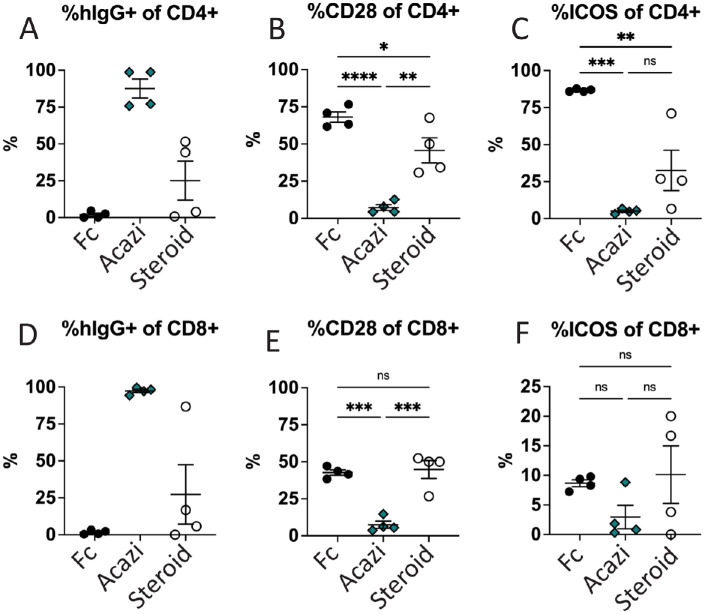
**Systemic acazicolcept provides blockade of CD28 and ICOS costimulatory receptors on ocular infiltrating T cells.** Engagement of acazicolcept with its targets on T cells was confirmed by the presence of the human IgG (hIgG) bound to ocular CD4+ and CD8+ cells. Blockade of target receptors demonstrated by low/absent receptor staining. (**A**) Percentage of hIgG+ CD4+ T cells. (**B**) Percentage of CD28+ or (**C**) ICOS+CD4+ T cells. (**D**) Percentage of hIgG+CD8+ T cells. (**E**) Percentage of CD28+ or (**F**) ICOS+ CD8+ T cells. Percentage per sample indicated by the symbol, mean of all samples indicated by the bar. Error bars indicate the SEM. Comparisons performed by one-way ANOVA with Tukey's multiple comparisons test. *P < 0.05, **P < 0.01, ***P < 0.001, ****P < 0.0001, ns = *P* ≥ 0.05.

Ocular T cells were also investigated for acazicolcept binding using anti-human IgG antibody to detect the presence of the human Fc fragment of acazicolcept by flow cytometry (Fig. 3). In animals receiving subcutaneous acazicolcept, the majority of CD4+ (88%) and CD8+ (97%) T cells were positive for the presence of acazicolcept Fc (Figs. 3A, 3D). This correlated with a statistically significantly lower percentage of CD4+ T cells positive for CD28 (7% vs. 68%, *P* < 0.0001) or ICOS (5% vs. 86%, *P* < 0.001) when compared to Fc control-treated animals (Figs. 3B, 3C). Corticosteroid-treated animals show spurious increases in the frequency of human hIgG+ CD4+ and CD8+ T cells (see Figs. 3A, 3D) likely an artifact of the very low absolute number of T cells present in these eyes. Previously, we demonstrated that when acazicolcept is bound to its targets on T cells, this interaction will block the binding of all commercially available anti-CD28 and -ICOS flow antibodies, including those used in this study.^40^^,^^41^ Thus, the absence or reduction of CD28 and/or ICOS detection by flow cytometry can indirectly reflect target binding by acazicolcept. Corticosteroid-treated animals also demonstrated statistically significantly smaller populations of CD4+ cells positive for CD28 (46%, *P* < 0.05) and ICOS (33%, *P* < 0.01) when compared to Fc controls, indicating that control of inflammation is also associated with decreased expression of these costimulatory receptors. Similar impacts on CD8+ T cell populations were also identified (Figs. 3E, 3F). Together, these data show that in eyes with uveitis the majority of CD4+, and to a lesser extent CD8+, T cells express the costimulatory receptors CD28 and ICOS, and treatment with acazicolcept provides significant blockade of these receptors on ocular infiltrating T cells.

### Impact of Treatment on Aqueous Cytokine Concentrations

To explore the impact of systemic and local treatment on ocular cytokines, the concentration of 27 cytokines was measured from the aqueous of treated eyes in both the systemic (Table 1, Supplementary Fig. S3) and local (Table 2, Supplementary Fig. S4) treatment studies. In animals treated systemically with Fc control, high concentrations (>1 ng/mL) of pro-inflammatory cytokines, such as IL-6 (mean concentration of 2257 pg/mL ± 210 pg/mL), IFNγ (2152 pg/mL ± 559 pg/mL), LIX/CXCL5 (1923 ± 503 pg/mL), IL-18 (1257 ± 210 pg/mL), and IP-10/CXCL10 (1060 ± 336 pg/mL) were detected. Treatment with acazicolcept or corticosteroids showed a trend toward decreasing concentrations of all cytokines measured, but no changes were statistically significant after controlling for multiple comparisons. Consistent with flow cytometry results showing decreased numbers of pathogenic CD4+ T helper subtypes (Th1 and Th17), aqueous concentrations of IFNγ were 40% to 50% lower in eyes from animals treated with systemic acazicolcept (1581 ± 417 pg/mL) and corticosteroids (1454 ± 84 pg/mL) when compared to aqueous from Fc control-treated animals. IL-17A was also lower in eyes from systemic acazicolcept- (347 ± 384 pg/mL) and corticosteroids (352 ± 446 pg/mL)-treated animals than in Fc control-treated animals (511 ± 184 pg/mL).

**Table 1.** Day 14 Mean Aqueous Cytokine Concentrations: Systemic Treatment Study

	Fc Control		Acazicolcept		Corticosteroid	
Cytokine	pg/mL	STD	pg/mL	STD	pg/mL	STD
**IL-6**	2257.0	210.4	2169.0	1531.0	2560.0	2496.0
**IFN-γ**	2152.0	559.9	1581.0	417.3	1454.0	81.4
**LIX (CXCL5)**	1923.0	502.5	1129.0	1141.0	712.8	605.6
**IL-18**	1445.0	463.4	1225.0	1144.0	1089.0	574.6
**IP-10 (CXCL10)**	1060.0	335.6	739.4	804.1	104.7	57.1
**Leptin**	968.9	314.5	470.9	169.4	479.7	241.1
**IL-5**	909.9	688.1	406.6	179.6	867.4	1101.0
**IL-1β**	619.3	244.1	760.4	1326.0	84.4	74.7
**IL-17A**	511.0	183.8	346.8	384.9	352.4	446.5
**VEGF**	499.1	468.3	219.4	300.4	166.5	171.4
**GM-CSF**	455.8	130.5	333.4	51.8	478.8	191.3
**IL-1⍺**	352.6	115.9	296.9	26.0	681.1	581.9
**IL-13**	327.8	69.3	260.7	32.8	300.7	65.9
**RANTES (CCL5)**	258.5	203.3	99.9	154.2	30.5	38.8
**IL-12 p70**	218.7	42.8	172.8	22.2	448.7	450.4
**IL-2**	183.0	51.0	128.5	34.4	458.1	565.9
**IL-4**	175.8	56.3	122.1	27.5	178.2	82.0
**MIP-2 (CXCL2)**	118.9	25.6	104.7	6.9	858.2	1256.0
**MCP-1 (CCL2)**	116.9	18.1	99.0	51.7	106.1	60.6
**IL-10**	113.3	28.1	91.1	22.2	362.4	447.5
**G-CSF**	54.0	19.0	39.0	9.1	98.7	101.1
**Eotaxin (CCL11)**	43.0	9.6	33.6	4.9	127.1	145.3
**Fractalkine (CX3CL1)**	41.8	13.2	31.7	9.2	76.5	77.5
**MIP-1⍺ (CCL3)**	35.6	20.1	20.2	10.3	35.2	34.2
**EGF**	18.4	12.2	13.0	4.4	21.4	7.9
**TNF⍺**	9.7	2.0	10.0	4.2	78.7	117.8

Average concentration of 5 pooled aqueous samples in pg/mL, standard deviation (STD).

**Table 2.** Day 14 Mean Aqueous Cytokine Concentrations: Local Treatment Study

	Fc Control		Acazicolcept		Corticosteroid	
Cytokine	pg/mL	STD	pg/mL	STD	pg/mL	STD
**IL-6**	54,740.0	42,414.0	10,709.0	5038.0	1702.0	2072.0
**IFN-γ**	10,595.0	2394.0	11,073.0	3056.0	5162.0	5117.0
**LIX (CXCL5)**	11,635.0	12,675.0	6020.0	7856.0	1691.0	2040.0
**IL-18**	4870.0	2863.0	7773.0	6022.0	5483.0	3292.0
**IP-10 (CXCL10)**	14,268.0	10,496.0	6906.0	7496.0	291.1	157.4
**Leptin**	5394.0	3022.0	2847.0	1857.0	1551.0	1021.0
**IL-5**	667.4	168.1	496.5	219.3	506.8	402.9
**IL-1β**	3077.0	4245.0	2245.0	3765.0	197.9	244.1
**IL-17A**	2195.0	2803.0	493.2	547.3	95.2	146.4
**VEGF**	1536.0	1825.0	1688.0	2556.0	355.7	228.9
**GM-CSF**	38.3	2.6	39.2	3.8	67.8	57.4
**IL-1⍺**	459.4	251.6	399.2	455.2	271.7	294.9
**IL-13**	359.4	300.2	7.9	0.5	47.8	80.1
**RANTES (CCL5)**	2248.0	1546.0	3088.0	4394.0	210.1	352.8
**IL-12 p70**	209.9	241.0	60.7	12.0	73.1	64.6
**IL-2**	198.1	135.9	179.9	205.2	201.3	268.1
**IL-4**	160.6	139.1	40.6	45.1	38.5	49.0
**MIP-2 (CXCL2)**	103.4	31.0	79.4	5.4	125.9	80.7
**MCP-1 (CCL2)**	7160.0	4463.0	4651.0	3791.0	1871.0	1957.0
**IL-10**	160.6	75.9	112.1	76.7	164.4	248.6
**G-CSF**	52.3	42.2	29.0	20.4	23.1	5.5
**Eotaxin (CCL11)**	101.4	45.3	57.8	34.1	41.8	30.4
**Fractalkine (CX3CL1)**	87.4	18.1	75.8	20.9	211.0	193.0
**MIP-1⍺ (CCL3)**	146.3	137.0	104.5	119.2	31.8	52.7
**EGF**	89.0	30.1	242.9	151.4	405.0	417.5
**TNF⍺**	31.5	18.3	38.7	26.8	28.6	23.2

Average concentration of five pooled aqueous samples in pg/mL, standard deviation (STD).

In the intravitreal injection study, treatment with acazicolcept or corticosteroids once again showed a trend toward decreasing the average aqueous concentration of all cytokines measured when compared to Fc controls. However, when we compared the aqueous concentration of a select cytokines such as IL-6, IFNγ, LIX/CXCL5, IP-10/CXCL10, and IL-17A between the systemic and local treatment studies, intravitreal injection produced concentrations that were many-fold higher than measured in the systemic treatment groups (see Table 2, Supplementary Fig. S4). These increases likely reflect additional local inflammation from the trauma of intravitreal injections on days 8 and 10. Together with the flow cytometry results, these cytokine results support a model in which systemic treatment with acazicolcept binds CD28 and ICOS, blocking effector T cell costimulation and activation and suppressing subsequent inflammatory cytokine production in the eye.

### Treatment With Systemic and Local Corticosteroids Causes Weight Loss in Rats

**Figure 4.**
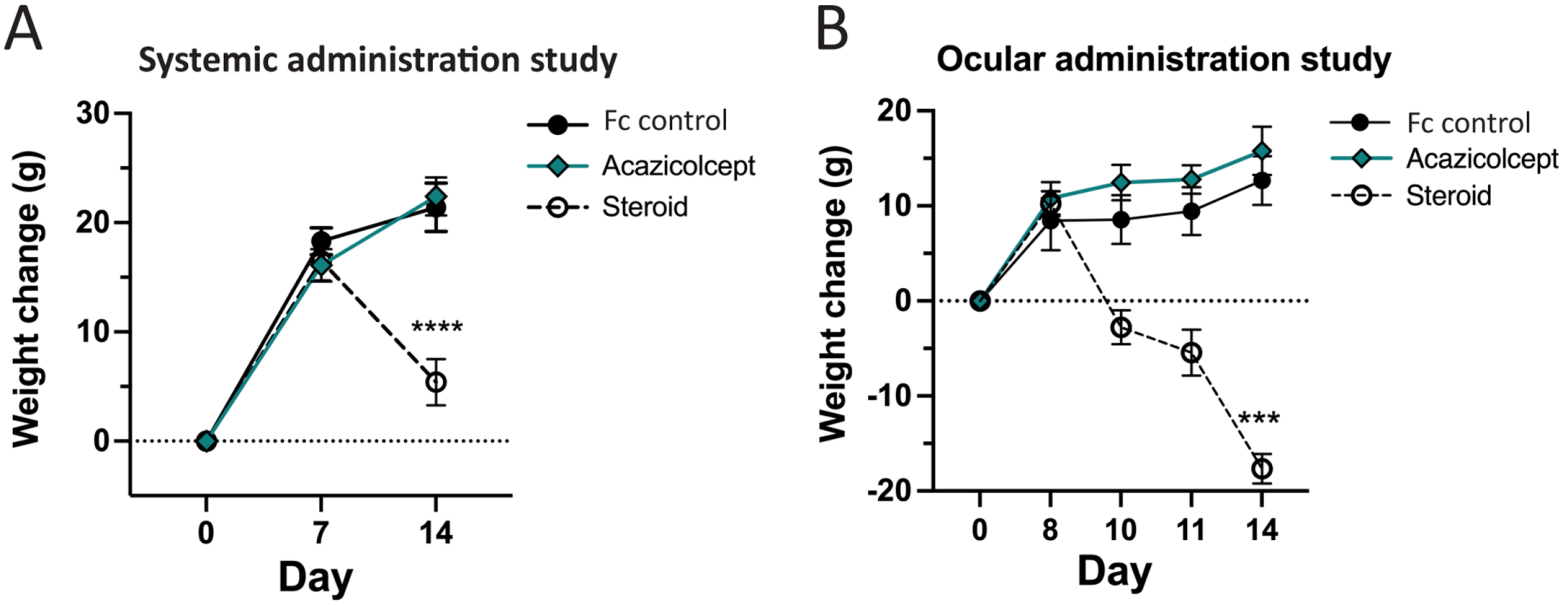
**Treatment with systemic and local corticosteroids demonstrates weight loss toxicity in rats.** Average weight change compared to baseline by treatment group for (**A**) the systemic treatment study (*n* = 30 total) and the (**B**) local treatment study (*n* = 27). Error bars indicate the SEM. Comparisons of weight change on day 14 performed by one-way ANOVA with Tukey's multiple comparisons test. Significance comparison of corticosteroid to Fc control and acazicolcept were the same and indicated once. ***P < 0.001, ****P < 0.0001.

Corticosteroids are highly effective treatments for uveitis, but their use is limited by systemic and local toxicities in humans.^9^^,^^10^ In rats, signs of toxicity from corticosteroids can include weight loss and death.^36^^,^^50^^,^^51^ To determine if corticosteroid treatment in this study was associated with signs of systemic toxicity, all animals were weighed at baseline (day 0), just prior to the start of test article treatment regimens (day 7 or 8), and at the end of the study. On the final study day, the body weights of animals treated with systemic corticosteroids (152.8 ± 10 gm, *P* < 0.01) or local corticosteroids (130.6 ± 5.2 gm, *P* < 0.0001) were statistically significantly lower than those in the acazicolcept treatment groups (systemic = 166.5 ± 6.3 gm and local = 161.0 ± 8.5 gm). In contrast, there was no difference in final weight between acazicolcept- and Fc control (systemic = 164.2 ± 4.9 gm and local = 159.4 ± 7.1 gm)-treated animals. Animals in all treatment groups initially gained the same amount of weight between day 0 and the start of treatment (Fig. 4), and by the end of the studies, Fc control- and acazicolcept-treated animals had gained an average of approximately 20 gm. In contrast, rats treated with one dose of systemic triamcinolone (0.8 mg/kg) only gained an average of 5 gm, and those receiving a total triamcinolone dose of 1.3 mg/kg from the 2 intravitreal injections lost an average of 15 gm from baseline. As all the rats should have gained an age-appropriate average of approximately 20 gm across the 2-week study period, these results show that corticosteroid treatment had a toxic impact on the general health of the rats; local administration did not prevent systemic corticosteroid distribution and the associated dose-dependent effects on inflammation and toxicity.

## Discussion

Here, we demonstrate the efficacy of acazicolcept, a dual CD28/ICOS antagonist, in controlling ocular inflammation in EAU. The EAU is a well-established model of autoimmune posterior and panuveitis and recapitulates key features associated with severe vision loss in humans, including vitritis, retinal vasculitis, neuroretinal inflammation, and photoreceptor loss. Central to disease pathogenesis in EAU is the development of antigen-specific T cells of the Th1 or Th17 subtypes. We found that systemic administration of acazicolcept prevented retinal damage and decreased ocular infiltration by Th1 and Th17 cells to a similar extent as treatment with corticosteroids. In contrast, local administration, by intravitreal injection, did not statistically significantly decrease inflammation when compared to Fc control-treated eyes. However, when compared to untreated fellow eyes of the same animal there was a clear trend toward improved inflammation scores in the eyes that received acazicolcept injection. Together, these findings indicate that dual T cell costimulation inhibition may be a disease-relevant therapeutic target in autoimmune uveitis and suggests acazicolcept could be explored as a novel alternative to corticosteroids for use in the treatment of patients with noninfectious uveitis.

Th1 and Th17 cells and their respective cytokines are central to the pathogenesis of noninfectious uveitis in animal models and humans. Whereas these pathogenic T cells function within the eye to coordinate disease activity, they are activated and expanded in extraocular tissues, such as cervical and intestinal lymph nodes.^52^^,^^53^ Our results suggest that inhibition of costimulation in these extraocular compartments is a more effective strategy than blockade within the eye. However, in this study, local and systemic administration are not directly comparable since animals treated intravitreally received approximately 100 fold less acazicolcept than those treated by subcutaneous injection. Whereas the initial pharmacokinetic (PK) study projected this intravitreal dose should provide therapeutic drug levels, these values were determined using uninflamed eyes. Previous studies have reported increased drug clearance rates in inflamed eyes, and this may have impacted our ability to detect a therapeutic effect in this study.^54^^,^^55^ It is possible that local anti-inflammatory effects could be improved with higher doses or formulations providing extended release. Furthermore, whereas acazicolcept binds and blocks rat CD28 and ICOS, it binds the rat targets approximately 1.6-fold less avidly than the human (data not shown). Another obstacle for detecting a benefit from local injection is the risk of inflammation exacerbation from local trauma to the crystalline lens. When compared to human eyes, rodent eyes are at much higher risk for inadvertent lens injury due to the large size of the lens relative to the overall eye size. In this study, one eye from the corticosteroid treatment arm was noted to have evidence of lens trauma by histology (see Fig. 1L, eye with the histology score of 22). Although no other eyes from any study group demonstrated visible signs of trauma, undetected trauma could have played a role in limiting our ability to detect a statistically significant benefit from local administration of acazicolcept. Studies in larger animal models, such as rabbits or nonhuman primates, could be pursued to clarify the potential effectiveness of local administration of acazicolcept in uveitis.

The study design for the systemic treatment trial only tested a single administration of acazicolcept 7 days after induction of EAU. This provided equivalent uveitis control as the depot injection of corticosteroid through day 13. However, on day 14, there was an uptick in the average inflammation score suggesting the therapeutic effect of acazicolcept may decline with time. Despite this slight increase in average clinical score, acazicolcept-treated animals were protected from developing severe inflammation (score of 3 or 4) over the entire study period. Evidence that uveitis control declined with time was also reflected in the aqueous cytokine results. Whereas aqueous IL-17 and IFNγ concentrations were on average lower in acazicolcept-treated animals when compared to Fc-control treated animals, individual animals that demonstrated an increase in clinical score between days 12 and 14 also had the highest concentrations of these cytokines on day 14. Together, these results suggest the treatment strategy of a single subcutaneous dose of acazicolcept effectively delayed uveitis onset, but that sustained control will require a higher subcutaneous dose, an alternate route of administration (intravenous versus subcutaneous), or repeat administration.

The current standard of care for uveitis treatment utilizes an approach tailored to inflammation severity and location.^12^ Initial disease control is achieved with appropriate doses of local or systemic corticosteroids followed by monitored corticosteroid taper or initiation of long-term immunomodulatory therapy (IMT). When IMT is required, a range of conventional corticosteroid sparing immunosuppressants, such as methotrexate, mycophenolate mofetil, azathioprine, cyclosporine, and tacrolimus, or biologic therapies are used as monotherapy or combined therapy following consensus treatment guidelines.^12^ The effectiveness of this approach in controlling ocular inflammation and preventing blindness is well established from large retrospective studies^17^^,^^20^^,^^21^^,^^56^ as well as from randomized controlled trials.^14^^,^^15^^,^^57^ Despite this range of treatment options, effective long-term control of uveitis remains a challenge for patients with uveitis refractory to current standard of care approaches, patients that develop drug-related side effects, or patients that experience complications of uveitis, such as cystoid macular edema despite ongoing therapy.^18^^,^^58^ New treatment options with alternative therapeutic targets are still needed. This study provides preclinical evidence for developing dual T cell costimulation blockade with acazicolcept as a novel therapy for use in patients with autoimmune (noninfectious) uveitis.

Previous studies investigating T cell costimulation blockade in animal models of uveitis have targeted the ICOS^25^^,^^36^^,^^38^ or CD28^59^ pathways independently. These prior studies showed that antagonism of costimulation tended to ameliorate uveitis but the degree of efficacy was variable and impacted by the timing of antagonist administration, the specific antagonist tested, and the animal model utilized. Our results with acazicolcept are consistent with these previous studies and further demonstrate the mechanistic importance of T cell costimulation to disease pathogenesis in autoimmune uveitis. A small number of studies in humans treated with abatacept, a US Food and Drug Administration (FDA) approved biologic targeting T-cell costimulation through interruption of the B7/CD28 interaction,^60^ suggest similar mechanisms are relevant in patients with uveitis. Although abatacept is not currently indicated for use in the treatment of uveitis, it is available for use in pediatric patients with refractory juvenile idiopathic arthritis (JIA). Children with JIA frequently develop co-morbid anterior uveitis that is chronic, difficult to control, and associated with multiple vision-threatening complications.^61^ In one study, abatacept was used as either primary biologic therapy after failing methotrexate or as a second-line agent for children failing conventional standard of care therapy with methotrexate and adalimumab.^62^^,^^63^ In this retrospective study of 35 patients with active JIA-associated uveitis, 17 (54.8%) were reported to achieve clinical remission after 12 months of therapy.^63^ In another study of pediatric patients with chronic anterior uveitis (any etiology) refractory to treatment with methotrexate and adalimumab, 5 of 10 (50%) of the children demonstrated remission of disease after 6 months of treatment.^64^ These results with abatacept support the rationale that therapies targeting T cell co-stimulation in the treatment of uveitis while highlighting the opportunity for improved disease control that could be provided by the dual pathway costimulation blockade provided by acazicolcept.

One concern associated with the use of immune modulation therapy is the risk of undesired or toxic side effects. No signs of toxicity were identified in the acazicolcept-treated animals in either the systemic or local treatment studies. In contrast, a significant dose-dependent weight loss was noted in the corticosteroid-treated animals. Rats treated with therapeutic doses of corticosteroids can demonstrate mild signs of toxicity, such as weight loss, or more severe impacts, such as failure to thrive and ulcerations of the gastrointestinal system.^36^^,^^47^^,^^51^ This is in contrast to humans, which manifest weight gain as a side effect of chronic corticosteroid therapy. Despite these species’ differences in side effect manifestations, it is significant to identify a similar benefit to uveitis control without the undesired side effects of corticosteroids. In clinical terms, the benefits of treatment must outweigh the risks. In our study, the rats treated with systemic acazicolcept demonstrated similar uveitis control to the corticosteroid-treated animals without the weight loss. Whereas this does not guarantee safety in humans, in the initial safety studies in healthy volunteers, acazicolcept was well tolerated.^65^ Additional studies in patients receiving long-term treatment will ultimately be required to establish the safety profile in patients with chronic autoimmune diseases.

One approach for avoiding potential side effects from systemic immune modulation is to deliver the effective IMT agent directly to the eye. The intravitreal approach has been used extensively for the delivery of corticosteroids in the treatment of uveitis and uveitic macular edema.^7^^,^^11^^,^^66^ Whereas this approach can be very successful for controlling inflammation, the significant risk of vision loss from complications, such as glaucoma and cataract, limits local therapy options for many patients. Alternative agents that control inflammation without the ocular risks of corticosteroids would be an important advance for the management of patients with uveitis. This approach has been tried previously with subconjunctival^67^^,^^68^ intravitreal^69^^,^^70^ injection of the immune modulating agent sirolimus. However, the first phase III intravitreal study failed to demonstrate sufficient effectiveness for FDA approval. Although multiple factors may have contributed to this outcome, one consensus opinion that emerged was that local administration may not be sufficient to modulate the contribution of systemic autoimmunity even in a localized disease process. Our results demonstrating significantly better uveitis control with systemic administration of acazicolcept is consistent with this consensus opinion. Despite this consideration, effective local treatment options to avoid the complications of local corticosteroids will continue to be important in the management of uveitic macular edema and could provide a novel option for improved control in patients with difficult-to-treat disease. Our findings that intravitreal injection of acazicolcept provided some benefit to decreasing clinical and histologic evidence of disease when compared to fellow eyes suggests that costimulation blockade should be explored further for possible benefit in local management of severe or refractory uveitis.

In summary, our results demonstrate that systemic treatment with acazicolcept significantly suppressed EAU in rats and was well-tolerated without the weight loss associated with corticosteroid treatment. These findings suggest that acazicolcept may be an effective alternative to corticosteroids for use in treatment of autoimmune uveitis in humans.

## Supplementary Material

Supplement 1

Supplement 2

Supplement 3

Supplement 4
