# Supplementary material for: Systemic Administration of Acazicolcept, a Dual CD28 and Inducible T cell Costimulator Inhibitor, Ameliorates Experimental Autoimmune Uveitis
Source: Transl Vis Sci Technol. 2023 Mar 28;12(3):27. doi: 10.1167/tvst.12.3.27 (PMC10064916; doi:10.1167/tvst.12.3.27)
Supplement: Supplement 1 [file tvst-12-3-27_s001.pdf]

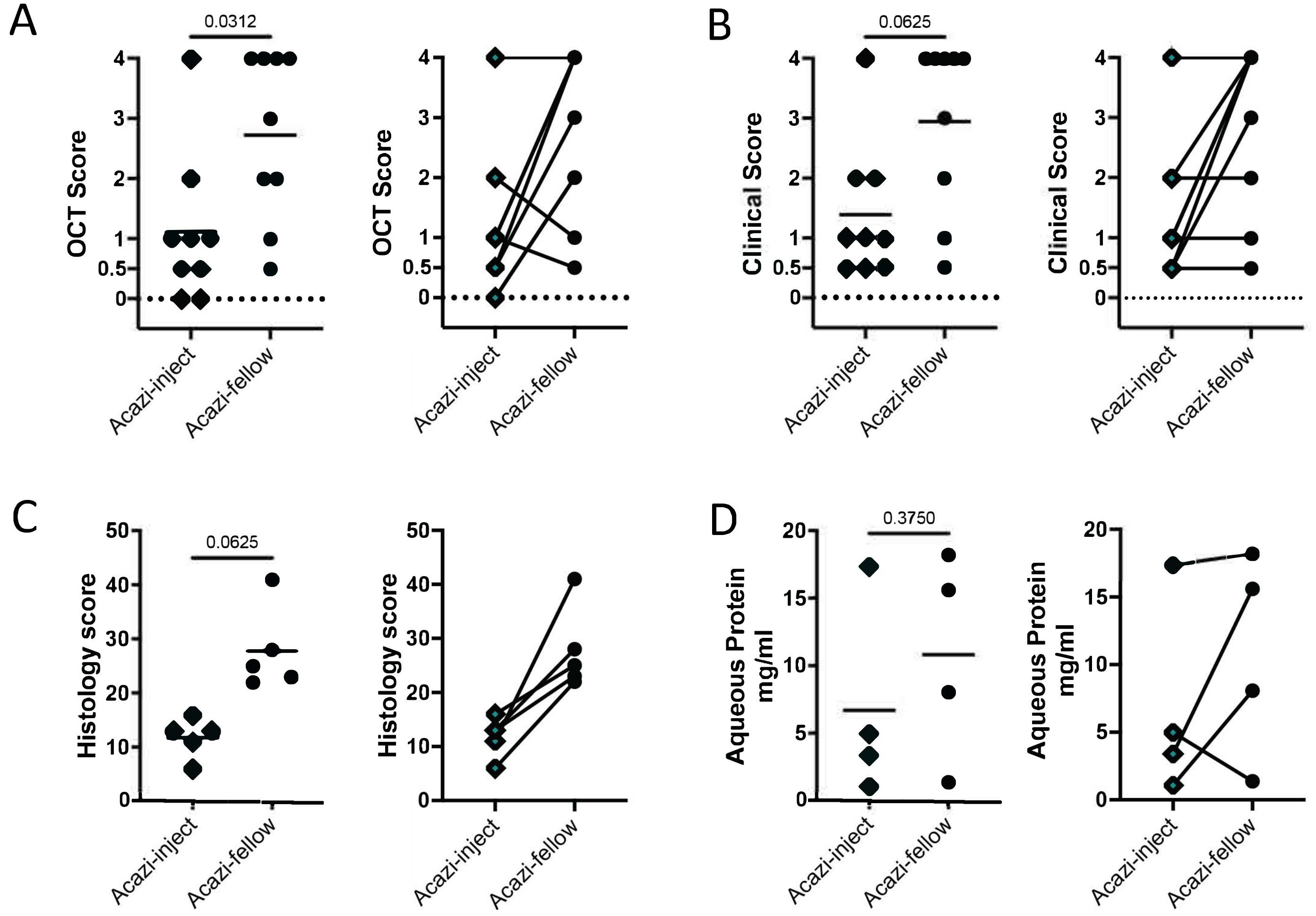

Supplemental Figure S1. Local treatment study subanalysis: Paired comparison of eyes injected with acazicolcept and uninjected fellow eyes. Average day 14 **(A)** OCT scores, **(B)** clinical scores, **(C)** histology score, and **(D)** aqueous protein concentration from injected (diamonds) and uninjected (circle) fellow eyes were compared by Wilcoxon signed rank test with p values indicated. OCT and clinical score were obtained for all animals (n=9) in the intravitreal injection study. Histology was performed for n=5 animals and aqueous protein concentration determined for n=4 animals. On the partner graph, eyes from the same animal are joined by lines. In A, OCT score for 7/9 (78%) animals shows that the eye injected with acazicolcept had a lower score than the untreated fellow eye. Only 7 lines are visible because two animals had paired scores of 1 & 4 (injected vs. uninjected eye) and two had paired scores of 0 & 2. OCT grading was performed by a grader masked to study day, treatment, and laterality. In B, only 8 lines are visible because two animals had paired scores of 1 & 4. Clinical score was performed by a grader masked to study treatment, but not study day or laterality.
