# Supplementary material for: Systemic Administration of Acazicolcept, a Dual CD28 and Inducible T cell Costimulator Inhibitor, Ameliorates Experimental Autoimmune Uveitis
Source: Transl Vis Sci Technol. 2023 Mar 28;12(3):27. doi: 10.1167/tvst.12.3.27 (PMC10064916; doi:10.1167/tvst.12.3.27)
Supplement: Supplement 2 [file tvst-12-3-27_s002.pdf]

Eyes From Fc Control-Treated Mice

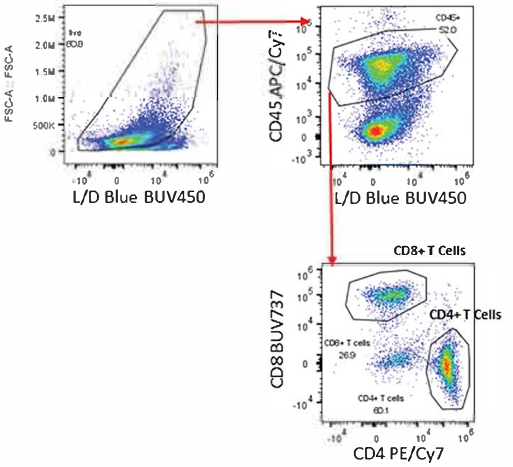

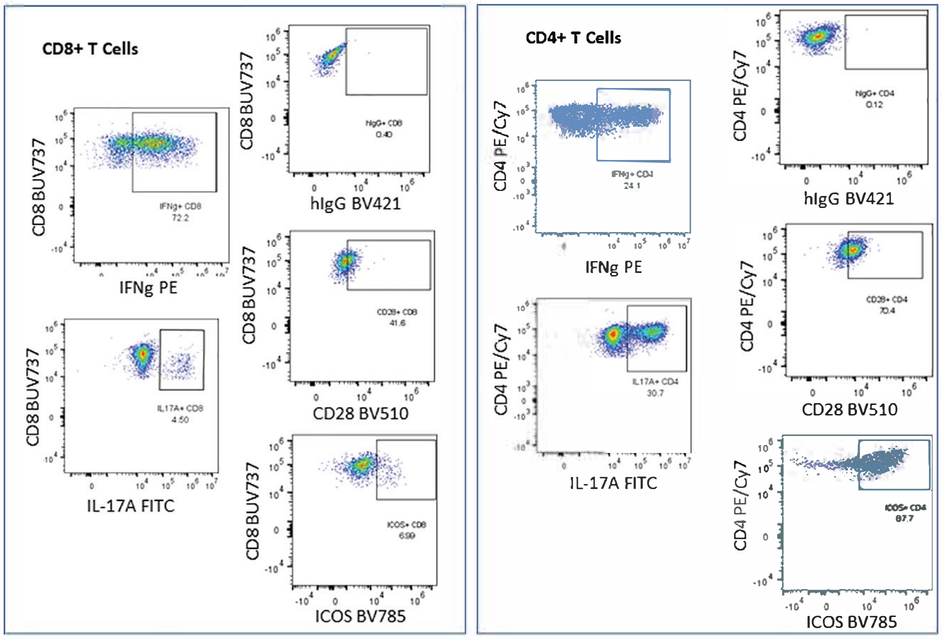

Eyes From Acazicolcept-Treated Mice

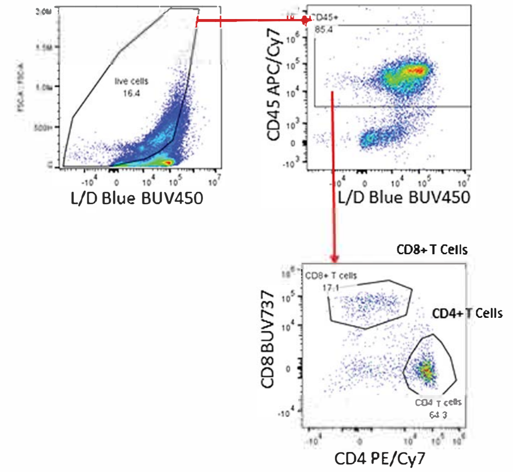

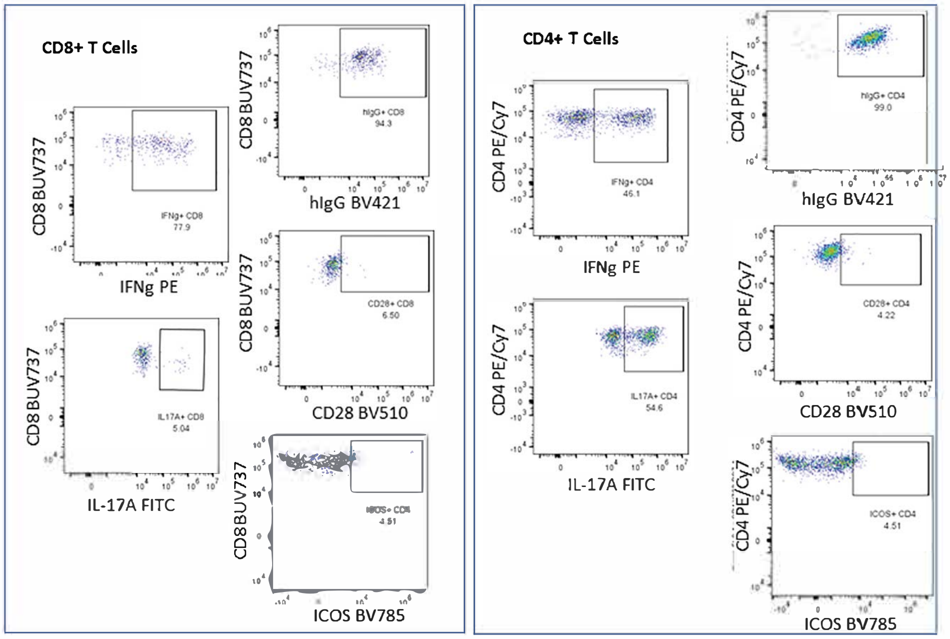

Eyes From Corticosteroid-Treated Mice

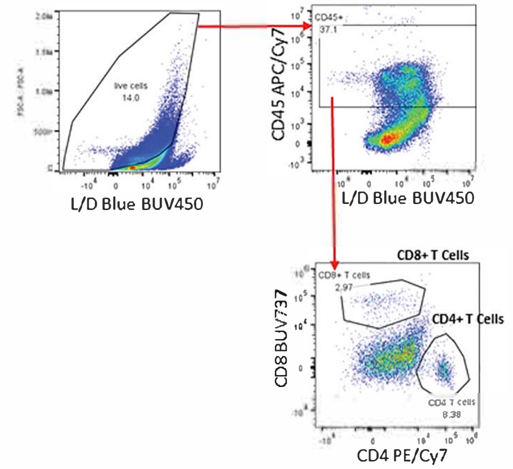

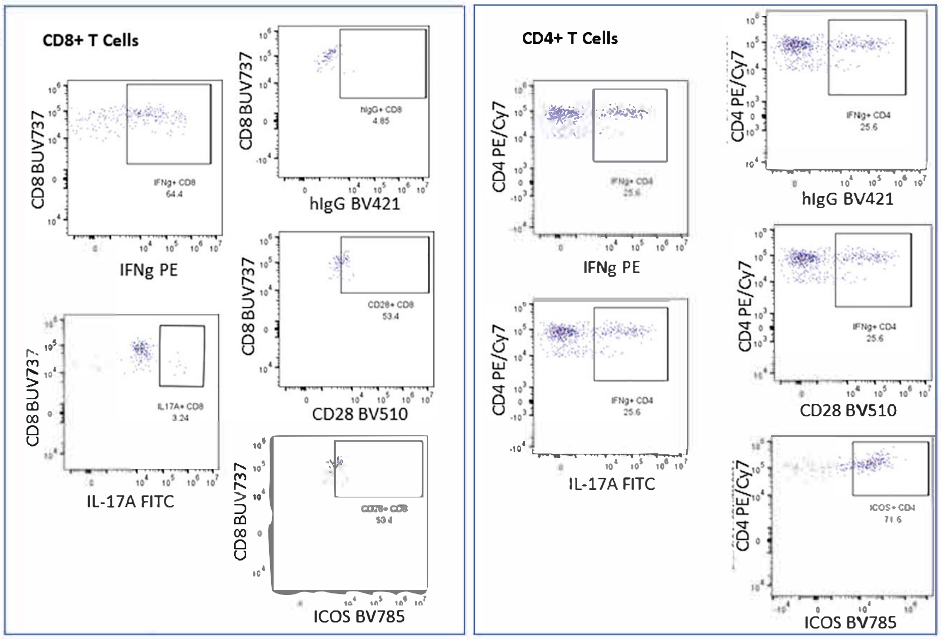

Supplemental Figure S2. Gating strategy for intracellular cytokine and cell surface targets from ocular samples. Flow cytometry was performed on intraocular contents of both eyes (pooled) from four animals in the systemic treatment study. Shown here are examples from one mouse in each treatment group: Fc-control (top), acazicolcept (middle), and corticosteroid (bottom). All samples were first gated for single, live cells, then CD45+ cells were further classified as either CD4+ or CD8+ T cells and characterized by expression of intracellular and cell surface markers as shown.
