# Supplementary material for: Systemic Administration of Acazicolcept, a Dual CD28 and Inducible T cell Costimulator Inhibitor, Ameliorates Experimental Autoimmune Uveitis
Source: Transl Vis Sci Technol. 2023 Mar 28;12(3):27. doi: 10.1167/tvst.12.3.27 (PMC10064916; doi:10.1167/tvst.12.3.27)
Supplement: Supplement 3 [file tvst-12-3-27_s003.pdf]

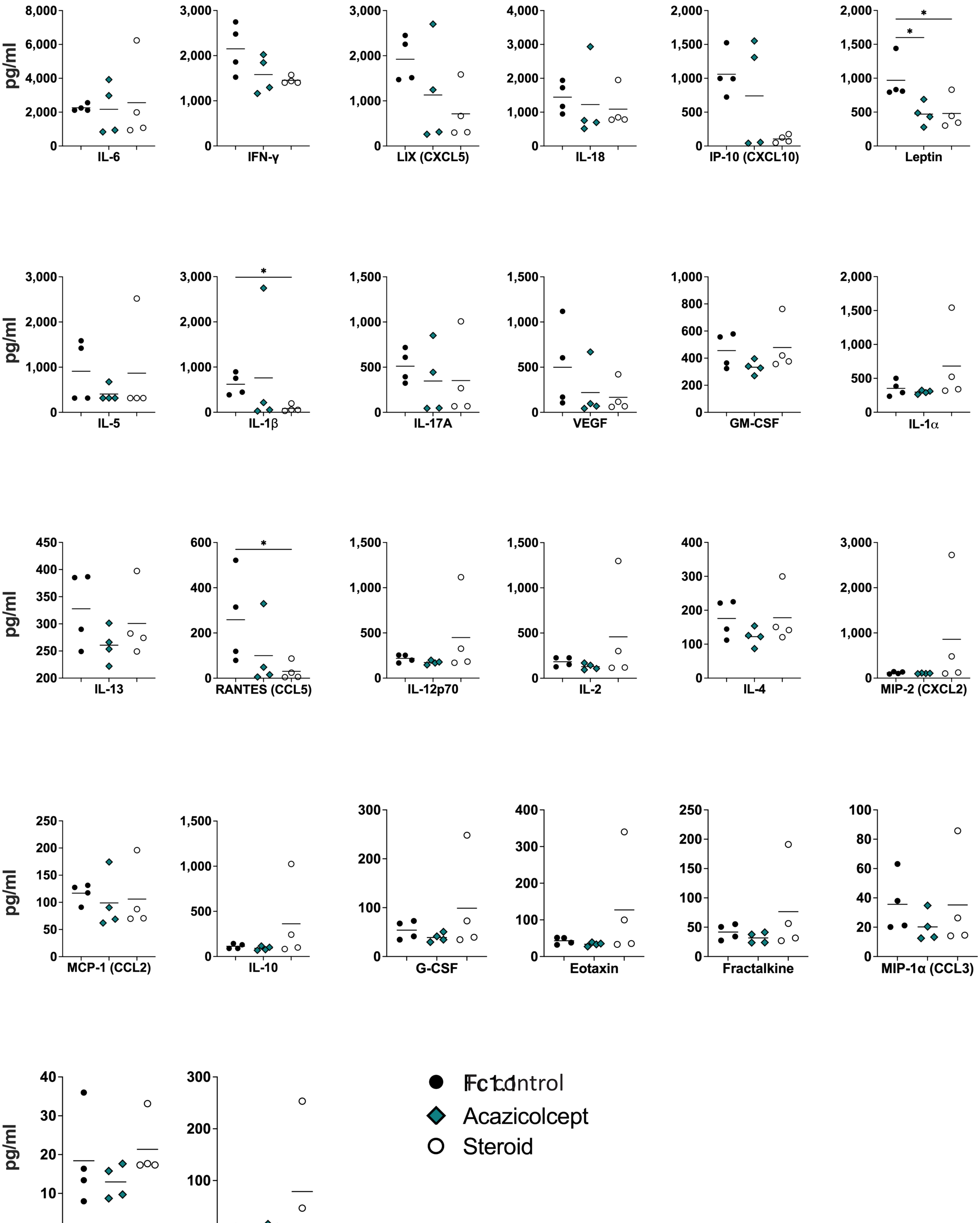

Supplemental Figure 1

Supplemental figure S3. Aqueous cytokine concentrations from eyes in the treatment study. Four rats per treatment arm were tested. Prior studies have demonstrated low to undetectable cytokine concentrations in naive rodent eyes.[49] Aqueous from both eyes was pooled into a single sample. Each symbol indicates the result from a single animal. Average concentration per treatment group is shown by the bar. Exploratory statistical analysis was performed for each cytokine using a Kruskal-Wallis test with uncorrected Dunn's post-hoc test. *$p<0.05$. Results shown are not corrected for the multiple comparisons required to test all cytokines.
