# Supplementary material for: Systemic Administration of Acazicolcept, a Dual CD28 and Inducible T cell Costimulator Inhibitor, Ameliorates Experimental Autoimmune Uveitis
Source: Transl Vis Sci Technol. 2023 Mar 28;12(3):27. doi: 10.1167/tvst.12.3.27 (PMC10064916; doi:10.1167/tvst.12.3.27)
Supplement: Supplement 4 [file tvst-12-3-27_s004.pdf]

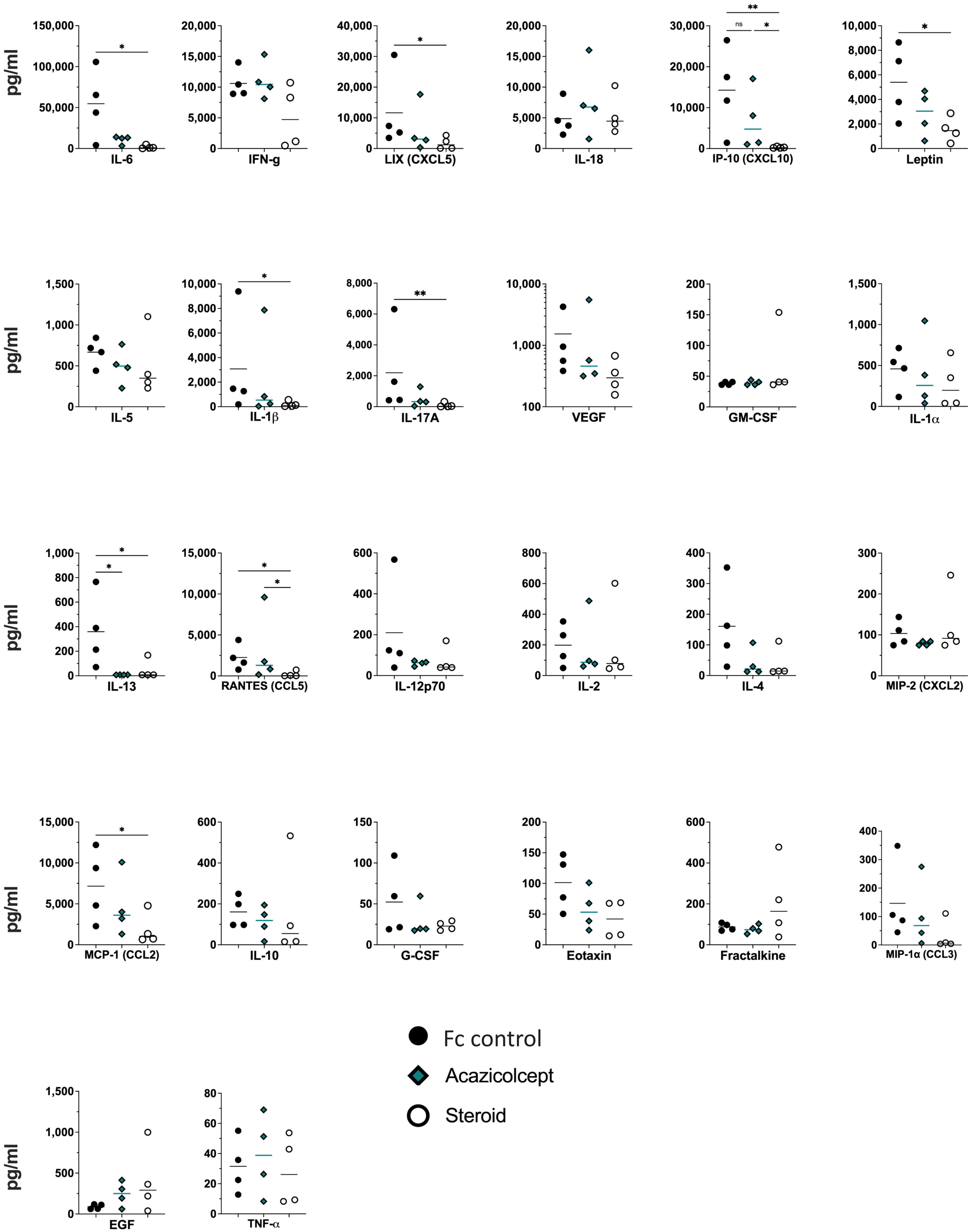
pg/ml
IL-6
IFN-g
LIX (CXCL5)
IL-18
IP-10 (CXCL10)
Leptin
IL-5
IL-1β
IL-17A
VEGF
GM-CSF
IL-1α
IL-13
RANTES (CCL5)
IL-12p70
IL-2
IL-4
MIP-2 (CXCL2)
MCP-1 (CCL2)
IL-10
G-CSF
Eotaxin
Fractalkine
MIP-1α (CCL3)
EGF
TNF-α
Fc control
Acazicolcept
Steroid

Supplemental Figure S4. Aqueous cytokine concentrations from eyes in the local treatment study. Four rats per treatment arm were tested. Aqueous from the injected right eyes was tested. Each symbol indicates the result from a single eye. Average concentration per treatment group is shown by the bar. Exploratory statistical analysis was performed for each cytokine using a Kruskal-Wallis test with uncorrected Dunn's post-hoc test. *$p<0.05$, **$p<0.01$. Results shown are not corrected for the multiple comparisons required to test all cytokines.
